# Contemporary Approach to Narrow Angles

**DOI:** 10.18502/jovr.v19i1.15443

**Published:** 2024-03-14

**Authors:** Wesam Shamseldin Shalaby, Rohit Reddy, Reza Razeghinejad, L. Jay Katz

**Affiliations:** ^1^Glaucoma Service, Wills Eye Hospital, Philadelphia, PA, USA; ^2^Tanta Medical School, Tanta University, Tanta, Gharbia, Egypt; ^3^Sidney Kimmel Medical College, Thomas Jefferson University, Philadelphia, PA, USA

**Keywords:** Glaucoma; Gonioscopy, Laser Peripheral Iridotomy, Lens Extraction, Narrow Angle

## Abstract

Glaucoma is the leading cause of irreversible blindness worldwide. Among all glaucoma types, primary angle closure glaucoma (PACG) affects approximately 23 million people worldwide, and is responsible for 50% of glaucoma-related blindness, highlighting the devastating consequences of this disease. The main mechanism of PACG is relative pupillary block. High-risk populations are female gender, Asian ethnicity, high hyperopia, short axial length, and a thick/anteriorly positioned lens. This review discusses the clinical diagnosis, classification, and management of patients with a narrow angle with and without intraocular pressure (IOP) elevation and glaucomatous optic nerve damage, including laser peripheral iridotomy (LPI), endocycloplasty (ECPL), lens extraction, and goniosynechialysis.

##  INTRODUCTION

Glaucoma is the leading cause of irreversible blindness worldwide and the second most common cause of bilateral blindness, following cataract.^[1,2]^ Primary-angle closure glaucoma (PACG) affects approximately 23 million people, and the number is expected to increase to 32 million by 2040.^[2]^ Although PACG is estimated to affect only 26% of all glaucoma patients, it is responsible for nearly 50% of glaucoma-related blindness worldwide.^[2,3]^ Compared with primary open-angle glaucoma-associated blindness, PACG carries a threefold increased risk of severe bilateral visual impairment.

The major mechanism of intraocular pressure (IOP) elevation and subsequent glaucomatous optic neuropathy in PACG involves the obstruction of the trabecular meshwork by the peripheral iris. While the trabecular meshwork may be anatomically and physiologically normal, the anterior position of the peripheral iris obstructs the flow of aqueous through the trabecular meshwork. The iridotrabecular contact may cause angle closure and IOP elevation by a transient apposition and acute IOP elevation, or by formation of peripheral anterior synechiae (PAS) and chronic impairment of the aqueous flow. In PACG, anatomic predisposition to pupillary block is the major identifiable abnormal finding on examination. Secondary angle closure glaucoma arises due to identifiable pathologic causes such as iris neovascularization, uveitis, intraocular mass, and an intumescent lens.

**Figure 1:**
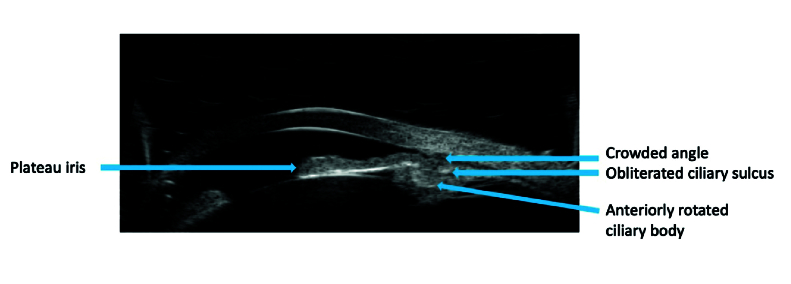
Ultrasound biomicroscopic features of plateau iris.

The risk of pupillary block is highest with a mid-dilated pupil where there appears to be maximum contact between the iris and the crystalline lens. The lack of aqueous flow creates a pressure gradient, causing the peripheral iris to bow forward and obstruct the trabecular meshwork. PAS may develop following prolonged or repeated iridotrabecular contact. Another possible mechanism of PACG is plateau iris configuration/syndrome, in which anteriorly rotated ciliary processes push the peripheral iris toward the meshwork, leading to angle closure [Figure 1]. This is seen more often in younger adults compared to pupillary block, however, in some of the PACG patients the component of plateau iris may lead to IOP elevation and PAS formation despite having a patent peripheral laser iridotomy (LPI).^[4]^ Additional mechanisms of angle closure include a thickened peripheral iris or prominent peripheral iris rollfilling the space between the trabecular meshwork and angle recess,^[5,6]^ particularly noticeable in dark conditions when the pupil dilates.^[7]^ A cross-sectional study utilizing anterior segment optical coherence tomography (AS-OCT) on the lasered contralateral eyes of patient with acute angle closure attack revealed iris volume increase. In contrast, control subjects, who had open angles, lost a substantial amount of iris volume with pupillary dilation.^[8]^ In open angles, the iris loses half of its area with dilation while in closed angle, the iris maintains its bulk by retaining water which increases the likelihood of angle closure.^[9]^

**Figure 2:**
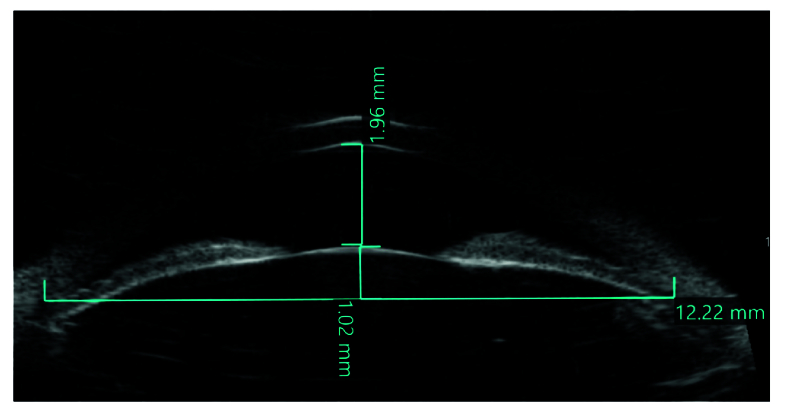
Ultrasound biomicroscopy image showing the lens vault (the distance between the anterior lens pole and the horizontal line connecting the scleral spurs), and anterior chamber depth (the distance between the central corneal endothelium and the anterior surface of the lens).

**Figure 3:**
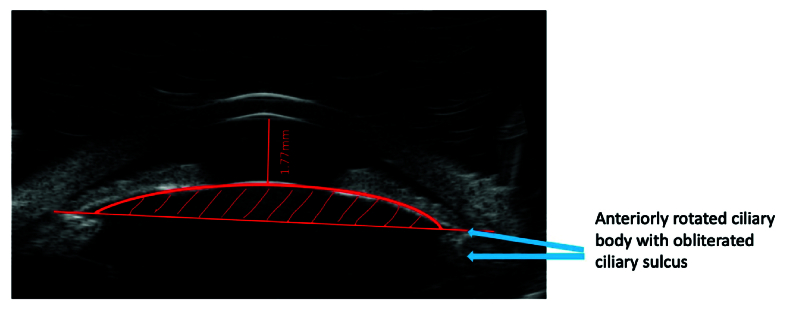
Ultrasound biomicroscopy image of a patient with closed angle showing a shallow anterior chamber, high lens vault (red area) and plateau iris (blue arrows).

Numerous risk factors play a role in the development of PACG, including increasing age,^[10]^ female gender,^[2,11]^ certain ethnicities (East Asian, Inuit Eskimo),^[2,11]^ family history and genetic predisposition,^[12,13,14]^ short axial length, thick/anteriorly positioned lens (high lens vault [LV]), flat cornea, and corneal diameter.^[15,16,17]^ LV is defined as the distance between the anterior lens pole and the horizontal line connecting the temporal and nasal scleral spurs [Figure 2], stands out as a parameter associated with angle closure on anterior segment imaging. Eyes with angle closure have a more crowded anterior segment due to the presence of a thicker and more anteriorly located lens causing high LV [Figure 3].^[17]^ Several AS-OCT studies have emphasized the strong association between high LV and angle closure across diverse ethnic populations.^[18,19,20]^

### Definition and Classification

According to the European Glaucoma Society Terminology and Guidelines,^[21]^ primary angle closure disease (PACD) is classified into three categories:

Primary angle closure suspects (PACS): iridotrabecular contact of 180–270º, IOP < 21 mmHg, normal optic disc, and no PAS.

Primary angle closure (PAC): PACS plus IOP > 21 mmHg and/or PAS, without any evidence of optic disc damage.

Primary angle closure glaucoma (PACG): PAC plus glaucomatous optic neuropathy.

Another classification of PACD is based on the clinical presentation and the timing or suddenness of onset into acute, intermittent, or chronic angle closure. In acute primary angle closure, the trabecular meshwork is suddenly blocked by the iris tissue, leading to acute IOP elevation.^[22,23]^ Symptoms include blurred vision, headache, ocular pain, nausea, and vomiting. Gonioscopy demonstrates appositional angle closure, although the view may be limited due to corneal edema, and with indentation the iris may not move back due to the crowded anterior segment. Other findings include elevated IOP; a mid-dilated, sluggish, and irregular pupil; shallow anterior chamber; cells and flare in the anterior chamber; and glaukomflecken (small anterior subcapsular lens opacities due to ischemic necrosis of lens epithelial cells).

Intermittent angle closure is characterized by intermittent IOP elevation due to transient angle closure. These patients may present with intermittent symptoms of angle closure (e.g., blurred vision, halo, ocular pain) and IOP may be normal at time of examination. Some of these patients may have been treated as a migraine case. Gonioscopy will demonstrate narrow angles with or without PAS.

In chronic angle closure glaucoma, damages to the optic nerve due to repeated and chronic IOP elevation and damages to the trabecular meshwork with or without PAS lead to visual field loss.^[23]^ Chronic angle closure glaucoma is generally similar to primary open-angle glaucoma in terms of presentation and prognosis. Therefore, these patients may initially present with severe visual field loss along with obvious optic nerve damage.

### Diagnosis

The narrow angle may be diagnosed with clinical examination and imaging studies.

#### Slit-lamp exam

The Van Herick method is a slit-lamp angle grading system that estimates the peripheral anterior chamber depth (ACD) by comparing it with the peripheral corneal thickness [Table 1].^[24]^ It may be used as a screening test to alert the examiner to narrow angles, however, it is not a substitute for gonioscopy.

#### Gonioscopy

Gonioscopy remains the gold standard tool for angle evaluation, however, it may be underutilized. Studies have found that a significant proportion of diagnosed open-angle glaucoma patients actually had angle closure.^[25,26]^ Other studies showed that gonioscopy is performed in < 50% of glaucoma patients and suspects.^[27,28]^

Gonioscopy lenses are divided into two major groups of indentation and non-indentation goniolenses. The non-indentation prototypes are Goldmann 1- or 3-mirrors goniolens. The diameter of this goniolens is larger than the corneal diameter, and doing any indentation indents the sclera and pushes the lens–iris diaphragm forward, causing a narrower angle. The indentation goniolenses include the Zeiss, Sussman, and Posner 4-mirror goniolenses. The diameter of the indentation goniolenses is smaller than the corneal diameter; thus, gentle, intermittent posterior pressure indents the central cornea and displaces the aqueous from the center to the periphery of the anterior chamber, mechanically deepening the angle and enabling better visualization of angle structures.^[29]^ This technique aids in determining the extent of PAS, thus differentiating appositional (appositional areas will open) from synechial (PAS will be exposed) angle closure. It can also differentiate plateau iris configuration from pupillary block, which are the main mechanisms of PACG. Corneal indentation during dynamic gonioscopy results in posterior movement of the peripheral iris in eyes with pupillary block, whereas in plateau iris configuration, the anteriorly rotated ciliary processes prevent peripheral iris movement, and a sine-shaped curve (double hump sign) on the peripheral iris is typically observed.

**Table 1:** Van Herick slit lamp angle grading system.


Grade 4	PAC ≥ 3/4 PCT	Angle is wide open
Grade 3	PAC > 1/4 and ≤ 1/2 PCT	Angle is mild narrow
Grade 2	PAC = 1/4 PCT	Angle is moderate narrow
Grade 1	PAC < 1/4 PCT	Angle is extremely narrow
Grade 0	PAC = 0	Angle is closed
	
	
PAC, peripheral anterior chamber; PCT, peripheral corneal thickness		

**Table 2:** Scheie gonioscopic grading system.


Wide Open	All structures visible
Grade I	Hard to see over iris root
Grade II	Ciliary body band obscured
Grade III	Posterior trabecular meshwork obscured
Grade IV	Only Schwalbe line visible
	
	

**Table 3:** Shaffer gonioscopic grading system.


Grade 4	45º to 35º angle	Angle is wide open
Grade 3	35º to 20º angle	Angle is wide open
Grade 2	20º angle	Angle is narrow
Grade 1	≤ 10º angle	Angle is extremely narrow
Slit	0º angle	Angle is closed
	
	

**Table 4:** Spaeth gonioscopic grading system.


**Iris insertion**		**Angular approach**	**Peripheral iris**				**Trabecular meshwork pigmentation**	
A	Anterior to Schwalbe's line	0º to 50º	r	Regular	b	Bowed anteriorly	0	No pigment
B	Between Schwalbe's line and scleral spur			1+	Minimal
C	Scleral spur visible	f	Flat	p	Plateau iris	2+	Mild
D	Deep with ciliary body band visible			3+	Moderate
E	Extremely deep with > 1 mm of ciliary body band visible	c	Concave		4+	Intense
	
	

**Figure 4:**
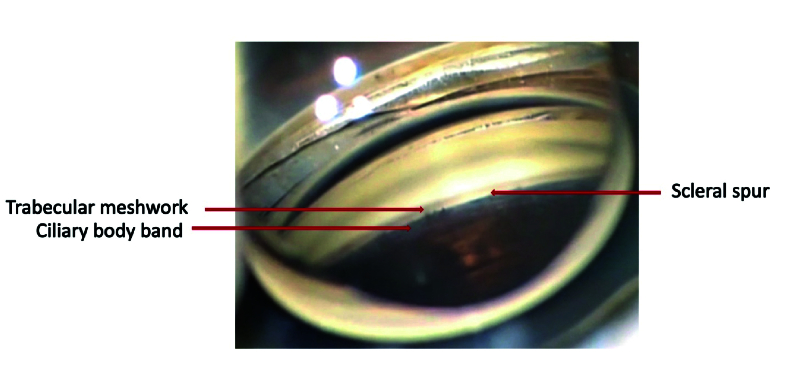
Gonioscopic view of a normal open angle.

**Figure 5:**
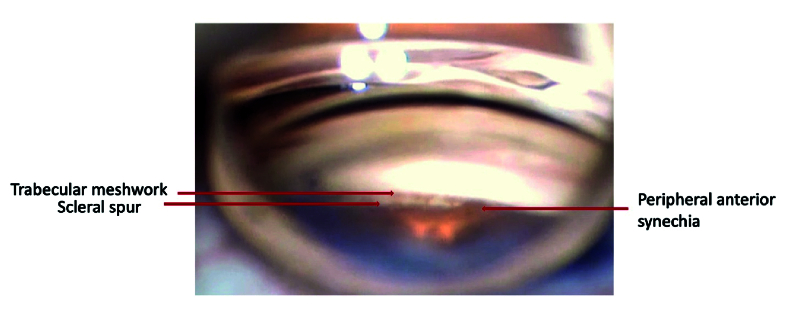
Gonioscopic view of open angle with area of peripheral anterior synechia.

In a normal open angle [Figure 4], starting at the root of the iris and toward the cornea, the following structures are observed:^[30]^ ciliary body band (gray to dark brown band that represents the area of iris insertion into the ciliary body); scleral spur (a prominent white line between the ciliary body band and trabecular meshwork); trabecular meshwork (a pigmented band anterior to the scleral spur with variable appearance depending on the amount and distribution of pigment deposition); and Schwalbe line (a fine ridge anterior to the meshwork representing the junction between the anterior chamber angle structures and the cornea with the terminal meeting of Bowmans and Descemet's corneal membranes). Example of PAS presentations is illustrated in Figure 5.

Since the first use of gonioscopy, a number of grading systems have been described to grade the anterior chamber angle. Scheie grading system is based on the structures visualized in Table 2;^[31]^ Shaffer grading is based on the width of the angle recess without indentation [Table 3].^[32]^ The Spaeth gonioscopic grading system is based on three variables following indentation gonioscopy: the location of the iris root insertion; the angle width, peripheral iris configuration, and the amount of the trabecular meshwork pigmentation [Table 4].^[33]^ The Spaeth grading system denotes parentheses to differentiate the apparent insertion versus the true anatomical insertion revealed by indentation gonioscopy. Indentation or dynamic gonioscopy is an important technique that should be performed using 4-mirror goniolenses rather than the classic Goldmann lens.

**Figure 6:**
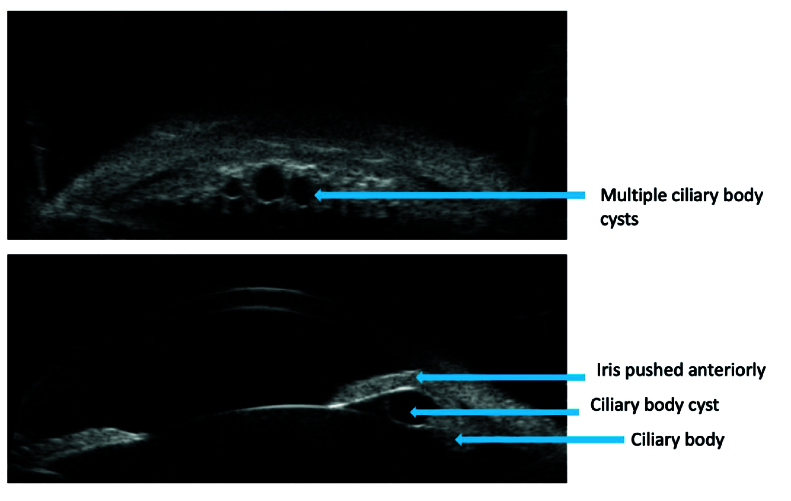
Ultrasound biomicroscopy image showing secondary angle closure due to ciliary body cysts pushing the peripheral iris anteriorly mimicking plateau iris.

#### Anterior segment imaging

While gonioscopy has long been the gold standard method in assessing angle anatomy, it has its own limitations. Primarily, it offers qualitative rather than quantitative information and requires a skilled examiner who is familiar with indentation gonioscopy and angle anatomy. The use of various grading systems and gonioscopic lenses introduces inter-examiner variability, casting doubt on the accuracy and reproducibility of angle grading.^[34]^ Anterior segment imaging has emerged as a valuable tool for providing cross-sectional angle pictures, playing an increasingly pivotal role in angle evaluation in recent years. The application of these devices has introduced new quantitative parameters for assessing angle morphology, with more objective and reliable data.

### Ultrasound Biomicroscopy (UBM)

High-frequency (35-100 MHz) Ultrasound Biomicroscopy (UBM), initially introduced by Pavlin and Foster in the early 1990s, is a non-invasive imaging modality that offers detailed two-dimensional grayscale images of the anterior segment.^[35]^ The procedure can be done using an immersion scleral shell (conventional immersion technique) or a disposable water balloon on the probe tip (ClearScan). Structures visualized with UBM includecornea, iris, anterior chamber angle, scleral spur, ciliary body, posterior chamber, anterior chamber, lens, and conjunctiva.

Prior studies have demonstrated a high level of agreement between UBM and gonioscopy in detecting iridotrabecular contact.^[36]^ Additionally, UBM provides valuable quantitative parameters of the anterior segment. These parameters include angle opening distance (AOD, the perpendicular distance between the trabecular meshwork at a point 500 µm anterior to the scleral spur, and the iris), angle recess area (ARA, the triangular area bound between the AOD line and the angle recess), ACD (the distance between the central corneal endothelium and the anterior surface of the lens), and LV [Figure 2].

In PACG, the AOD and ACD are lower than the figures in open angle, providing valuable diagnostic information. UBM is particularly useful in confirming the presence of plateau iris, as it can image the ciliary body and posterior chamber, structures beyond the reach of clinical gonioscopy [Figure 1].^[37]^ Additionally, it can be used in eyes with opaque media, for evaluation of lens, secondary causes of angle closure, for example, iridociliary cysts and masses [Figure 6]. However, UBM has its limitations – requiring a skilled operator, direct contact with the eye which can be cumbersome, and the potential for a false widening of angle when performed in supine position.^[38]^

### Anterior Segment-Optical Coherence Tomography (AS-OCT)

Optical coherence tomography (OCT) is an optical imaging modality utilizing low coherence interferometry to obtain static cross-sectional images of biological tissues. Its application in the anterior segment (AS-OCT) was first reported in 1994.^[39]^ Unlike UBM, it a non-contact imaging technology with a fast high-resolution image acquisition and does not require experienced operators. As a non-contact modality being performed in sitting position, it is comfortable for patients and can capture the real-time changes in angle morphology in response to rapid alteration of room illumination. However, it is not a suitable anterior segment imaging in eyes with media opacity, and cannot visualize the structures behind the iris. While AS-OCT can effectively discern mechanisms of angle closure, such as pupillary block and exaggerated LV, the diagnosis of posterior mechanisms like plateau iris and iridociliary lesions needs imaging by UBM.

Structures typically visualized with AS-OCT includecornea, iris, anterior chamber angle, scleral spur, ciliary body (poorly), posterior chamber (poorly), anterior chamber, lens (partially), and conjunctiva. Standard AS-OCT parameters include AOD (500 or 750), ARA (500 or 750), ACD, LV, trabecular–iris space area (TISA 500 or 750, trapezoidal area bounded by the AOD 500 or 750, the anterior iris surface, the inner corneo–scleral wall, and the perpendicular distance between the scleral spur and the opposing iris), trabecular–iris circumference volume (TICV 500 or 750, the integrated volume of the peripheral angle derived from TISA taken at 256 locations in the angle), iris area (Iarea, the cross-sectional area of the full length of the iris from the scleral spur to the pupil margin), and iris volume (the entire iris volume that is calculated from the summation of eight partial volumes mathematically estimated from four iris cross-sectional areas at 45º intervals).

Initial studies reported that AS-OCT exhibited high sensitivity in detecting angle closure compared to gonioscopy,^[40]^ with a tendency to identify more closed angles, especially in the superior and inferior quadrants.^[41]^ However, a subsequent study, that compared the accuracy of Visante and Cirrus AS-OCT with gonioscopy conducted by trained examiners, reported only slight to fair agreement between OCT and gonioscopy angle closure detection. Additionally, the study demonstrated less consistency between OCT machines than among clinician examiners. The limited ability of AS-OCT to diagnose angle closure was attributed to challenges in identifying angle structures, with the scleral spur identified in only 56% and 50% of quadrants with Visante and Cirrus OCT, respectively.^[42]^

Newer models, using swept source OCT have demonstrated reproducible and quantifiable information on the extent of PAS.^[43]^ Additionally, the in-built software analysis can assess the extent of iris–trabecular contact (ITC index), and was found to have moderate agreement and good diagnostic performance for angle closure compared to gonioscopy as the reference standard.^[44]^ Another reliability analysis found that the parameters with the best discriminative ability for detecting narrow angles were AOD 750 inferiorly, TICV 500, and TICV 750.^[45]^

### Gonio-photographic Systems 

To address the challenge of limited recordability associated with clinical gonioscopy, various systems of gonio-photography have emerged in recent years. Among these systems are two commercially available devices: EyeCam (Clarity Medical Systems, Pleasanton, CA) and NIDEK automated gonioscope (NGS-1, NIDEK Co, Gamagori, Japan).

EyeCam is an advancement of RetCam, initially designed to capture wide-field fundus photos. This portable handheld device, like gonioscopy, necessitates contact with the eyeball. The technique provides a direct, color image of the angle with excellent optical quality. Earlier reports showed that EyeCam correlated well with gonioscopy for detecting angle closure. It captured clear images of angles in 98.8% of participants.^[46]^ Further studies demonstrated an overall sensitivity of 76.2% and specificity of 80.9% in detecting gonioscopic angle closure, showing good agreement with gonioscopy.^[47,48]^ The cost of the device and taking longer time to capture the images compared to gonioscopy are among the limitations of EyeCam. Placing the patient in supine position for imaging and the bright illumination, delivered via a fiberoptic cable, may falsely open the angle. Discerning angle structures with EyeCam may be challenging when the trabecular meshwork is lightly pigmented.

The NGS-1 automated gonioscope has the capability to acquire comprehensive 360º gonioscopic images of the angle in < 60 sec per eye. This is achieved through the utilization of a 16-face multi-mirror optical gonioprism and an integrated image sensor. With the incorporation of an inherent rotator unit, a colored circumferential image of the iridocorneal angle is obtained in a single examination. The learning curve for proficiency is approximately one week, primarily involving the acquisition of skills related to aligning the patient's eye with the camera.^[49]^ Although the NGS-1 use is approved in other countries, it is considered investigational in the United States because it is not currently approved by the Food and Drug Administration. Prior studies have shown fair to moderate intra- and inter-observer agreement for NGS-1 angle images.^[50,51,52]^ Limitations of the NGS-1 include the inability to perform dynamic (indentation) gonioscopy and insufficient depth of view for angle grading.

### Machine Learning

A recent advancement in angle imaging involves the qualitative and quantitative analyses of UBM and AS-OCT images through deep learning algorithms. These algorithms utilize raw images obtained directly from the device along with the input of anterior segment parameters, facilitating angle closure detection and classification.

Preliminary results using fully automated UBM deep learning device demonstrated high accuracy and good consistency with the manual measurements.^[53]^ Further studies showed sensitivity and specificity for angle closure of 98.7% and 97.4%, respectively.^[54]^ Compared to the ophthalmologists angle grading, recent models achieved high accuracy in classification.^[55]^

Utilizing AS-OCT input, automated software systems have demonstrated a high accuracy in classifying anterior chamber angles (including open angle, narrow angle, and angle-closure grades),^[56]^ as well as excellent performance in predicting plateau iris.^[57]^ Although earlier models required manual identification of the scleral spur and accurate segmentation verification, recent systems have the ability to perform absolute automated analysis with annotation of scleral spur and segmentation of anterior segment structures like human experts, in both open and closed angle.^[58]^

### Management

#### Laser peripheral iridotomy (LPI)

*Indications*

Ever since the 1993 landmark study by Wilensky that reported 6% of angle-closure suspects developed angle closure over a mean of 2.7 years,^[59]^ ophthalmologists have viewed narrow angles as best managed prophylactically with LPI. LPI alleviates pupillary block by allowing the aqueous to pass directly from the posterior chamber into the anterior chamber through the iridotomy and bypassing the pupil.

Several studies have examined the prophylactic effect of LPI on conversion from PACS to PAC or PACG. The Chinese Zhongshan Angle-closure Prevention (ZAP) trial randomized 889 PACS participants into treatment and control by the eye, such that one eye randomly received LPI while the other eye was the control.^[60]^ The endpoint was conversion to PAC or development of acute angle closure attack over a follow-up duration of six years. The primary outcome event occurred in 19 (2.1%) treated versus 36 (4.1%) untreated eyes (*P* = 0.004). The ZAP trial found a very low conversion rate of 4% in the untreated eyes; that translates to < 1% per year, which was far less than expected. Although the study did find that LPI reduced the risk by approximately 50%, (4–2%), prophylactic LPI was not recommended in PACS for this Chinese population given the low overall conversion to PAC and absence of immediate threat to vision. The 14-year outcomes of the ZAP trial were published recently.^[61]^ A total of 390 LPI-treated eyes and 388 control eyes were lost to follow-up, and 70 LPI-treated eyes and 54 control eyes received cataract surgery before the 14-year visit or conversion to PAC. At the 14-year visit, 33 LPI-treated eyes (4.6 eyes/1000 eyes/year) and 105 control eyes (13.6 eyes/1000eyes/year) developed PAC. After adjusting for the inter-eye correlations, the difference in conversion to PAC between the treated and untreated eyes remained significant (*P* < 0.01).

The similar Singapore Asymptomatic Narrow Angles Laser Iridotomy Study (ANA-LIS) enrolled 480 PACS patients.^[62]^ Similar to ZAP, ANA-LIS randomly treated one eye with LPI, and the contralateral eye was the control. The findings from this study were similar to the ZAP trial in terms of the benefit of performing LPI. LPI reduced the risk of conversion by 45%. The major difference was the higher conversion to PAC (about 10% over five years). The study found that older participants and eyes with higher baseline IOP were more likely to convert to PAC. The number needed to treat to prevent one case of PACS converting to PAC or PACG at five years was 22 and 103, respectively.

Both the ZAP and ANA-LIS studies focused on patients of Chinese ethnicity; thus, the results are not generalizable to other ethnicities. As it relates to the United States, Yoo et al presented a large retrospective case study to analyze the conversion rate from PACS to PACG over six years. Among the 3985 patients meeting the inclusion criteria, 459 (11.52%) converted to PACG within the study period. The conversion rate was stable at 3.5% per year after the first six months of diagnosis. In the Cox proportional hazards model, age > 70 years was positively associated with conversion, while cataract surgery had a protective effect against diagnostic conversion. The authors concluded that the annual risk of diagnostic conversion from PACS to PACG is relatively low and highlighted the need for improved clinical methods to identify patients at higher risk for PACG.^[63]^

Patients on systemic medications with anticholinergic/mydriatic effect that may precipitate an episode of acute angle closure, regular pupillary dilation in patients with diabetes or age-related macular degeneration, dementia or any mental illness that may hamper angle closure symptoms and delay detection, and problems associated with poor access to healthcare are situations in which closer observation of PACS is appropriate. Additionally, patients with symptoms of intermittent angle closure attacks, family history of glaucoma, or ≥ +3.00 D of hyperopia are difficult to leave untreated because they seem to be at higher risk of developing PAC or PACG. Moreover, observing a narrow angle patient who converts to acute attack of glaucoma may result in permanent visual loss and, in addition, invite legal consequences for the care provider. A careful benefit to risk discussion is advisable with PACS patients who may prefer undergoing LPI rather than merely observing for progression with more frequent monitoring.

#### Technique ${\;}^{\left[64,65\right]}$

Generally, LPI can be performed with the neodymium: yttrium-aluminum-garnet (Nd:YAG) laser. In cases of dark irides or patient on antithrombotic therapy, a combination of the argon laser and the Nd:YAG laser works well to lower the total energy needed and minimize the risk of significant pigment dispersion and bleeding, which can interfere with completion of the procedure and increase the risk of an IOP spike.

A topical anesthetic (proparacaine 0.5%) is instilled as well as pilocarpine 1–2% to induce miosis, stretch the iris taut, and facilitate perforation. Contact lens (e.g., Abraham +66 D, Wise +103 D or CGI©LASAG CH lens) is used to improve the view and stabilize the eye. The plane of the contact lens must always be oriented parallel to the iris plane, and the aiming beam should be centered within the center of the magnifying button and must always be in sharp focus prior to laser activation.

Different laser settings are employed depending on the device used, iris pigmentation, and the physician's preference. In patients with blue, green, or light brown irides, the recommended settings are a power of 4–8 mJ and 1–3 pulses/burst. In patients with dark brown irides or on blood thinners, the iris stroma is initially thinned with the heat generating argon laser that coagulates any potential iris vessels and prevents bleeding. Then, the Nd:YAG laser is employed to finish the full thickness iridotomy. The recommended argon laser settings are an aiming beam spot size of 200 microns, laser power of 500–1000 mW, and duration of 0.05–0.1 sec. A small (150–200 micron),^[66]^ peripheral, and completely patent iridotomy is the ideal result.

To detect any IOP spike following LPI, IOP should be checked within 30–90 min following the procedure. Additionally, the eye should be pretreated with topical apraclonidine (0.5–1%) or brimonidine (0.1–0.2%) to decrease the risk of IOP spikes.^[67,68]^ Topical steroids are used for five to seven days, and during follow-up the patency of iridotomy, ACD, and angle opening are evaluated.

#### Location

The iridotomy site should be in the peripheral third of the iris. A crypt or a thinned area of the iris is an ideal place for laser application. The area between 11 and 1 o'clock was common practice, where the iridotomy is superior and fully covered by the lids. Visual symptoms of glare and ghost images following LPI may be more likely to occur in patients who had partially or fully exposed iridotomies versus those in whom the iridotomy was completely covered by the lid.^[69]^ However, a randomized, prospective, single-masked clinical trial (*N* = 208) reported new-onset linear dysphotopsia in 10.7% of eyes with superior LPI versus 2.4% of temporal LPIs (*P* = 0.002).^[70]^ Significantly higher visual symptoms with superior location of the iridotomy may be related to the lid margin–tear film meniscus that causes a prismatic effect to refract more rays light toward and through the iridotomy to enter the eye. This in turn would cause more prominent diffraction rings, interference fringes, and ultimately glare.^[71]^

Another multicenter, randomized, prospective, single-masked trial was conducted in India on the effect of LPI location on dysphotopsia symptoms of 559 patients.^[72]^ The visual disturbances were evaluated two weeks after LPI using a dysphotopsia symptom questionnaire described by Spaeth et al.^[69]^ Following LPI, 8.9% of all patients reported new visual symptoms; the most common were linear dysphotopsia, glare, and blurring. Although superior LPI was associated with lower incidence of new onset dysphotopsia compared with nasal/temporal LPIs (8.4% vs. 9.5%), the difference was not statistically significant (*P* = 0.7). In the multivariate logistic regression analysis, neither LPI location and size, nor total laser energy predicted higher odds of postoperative dysphotopsia (*P* > 0.1 for all).

The ZAP trial also reported the impact of LPI on visual symptoms in a separate cohort derived from the original randomized trial using an external control group (no LPI) to identify LPI parameters influencing the visual symptoms.^[73]^ At month 18 following LPI, treated subjects underwent digital iris photography and photogrammetry (mapping and measuring the dimensions based on the digital images) to characterize the size and location of the LPI. Measurement of retinal straylight and visual symptoms questionnaire was completed in 217 lasered and 250 controls. Straylight score did not differ among treated versus untreated eyes, nor among LPI partially covered versus totally uncovered by the eyelid. Prevalence of subjective glare did not differ significantly among participants with totally covered versus partially covered versus totally uncovered LPI. In regression models, only worse cortical cataract grade (*P* = 0.01) was associated with straylight score, and no other factors were associated with subjective glare. The LPI size and location were not associated with straylight or subjective symptoms. The majority of visual symptoms were found to resolve after six months of follow-up which may indicate that patients adapt or learn to ignore them.^[74]^ Patients may be reassured that improvement of symptoms may occur spontaneously over time. However, treatment may be needed in patients with persistent visual symptoms. Initial treatment includes opaque contact lenses that were shown to successfully eliminate the symptoms.^[75]^ Additionally, lamellar intrastromal corneal tattooing has been reported for treatment of persistent visual symptoms in patients who are intolerant to contact lenses. The procedure has been applied in eye-bank eyes with simulated iris defects to explore the practicality of the technique,^[76]^ and in case reports for treatment of patients with visual symptoms following LPI.^[71,77]^

#### Complications

Generally, LPI is safe and well tolerated by most patients. In the Philadelphia Glaucoma Detection and Treatment Project, 132 patients who underwent bilateral, same-day LPI, all tolerated the treatment without serious complications.^[78]^ Below are some of the common complications of LPI.

#### Transient IOP spikes

IOP spike is the most commonly reported complication following LPI. It is typically transient, occurring most frequently in the first 4 hr after laser, and lasts < 24 hr.^[64,79]^ Its occurrence seems to be secondary to the obstruction of the trabecular meshwork by released blood and iris pigment. Other possible causes include non-pupillary block angle closure mechanisms such as plateau iris syndrome, inflammation, or performing laser on patients with extensive PAS. Use of perioperative topical alpha agonists (e.g., apraclonidine or brimonidine) was found to be prophylactic against IOP spikes and is now a common practice.^[67,68]^

#### Hyphema

Hyphema occurs due to bleeding from the iridotomy site given the photodisruptive, nonthermal nature of Nd:YAG laser. It is usually mild and can be stopped by light pressure applied to the eye with the iridotomy lens. It has been found that use of antithrombotic therapy did not increase the incidence or severity of hyphema (*P* = 0.14) in a prospective controlled trial by Golan et al.^[80]^ Therefore, it was not recommended to discontinue these medications before LPI.^[79]^ However, massive hyphema has been reported following LPI in a patient using dual antiplatelet therapy.^[81]^ The authors argued that Golan's study included no patients on a dual antiplatelet therapy. Moreover, when they repeated calculations based on the data published by Golan et al, and using one-tailed Z score, there was a significant increase in incidence of bleeding in patients who did not stop antithrombotic therapy (*P* = 0.046). So, patients on antithrombotic therapy should be counseled on the risk of bleeding. Use of argon laser before Nd:YAG laser in patients on antithrombotic therapy can minimize the risk of bleeding. Consultation regarding whether temporarily discontinuing anti-coagulants is risky, with the patient's family physician, is strongly recommended.

#### Temporary anterior uveitis

Temporary anterior uveitis is postulated to be due to the release of prostaglandins and inflammatory mediators.    It is usually mild and is successfully treated with topical steroids. In rare situations, sterile hypopyon or posterior synechiae may develop.^[82,83]^ Prednisolone acetate 1% for one week after LPI is the typical treatment for anterior uveitis.

#### Cataract progression

The risk of a cataract has been reported at follow-up periods ranging from one to six years post LPI. Possible mechanisms include direct lens tissue disruption by the Nd:YAG laser, heat buildup by the argon laser, or mild inflammatory and metabolic changes that could accelerate cataract formation. The risk increases if the iridotomy site is close to the pupil.^[84,85,86]^

#### Iridotomy closure

Closure of the iridotomy site may occur in the early postoperative period (two weeks) in 1% of patients, which may increase to 20% within the first six months.^[78,79,87]^ The mechanism is thought to be accumulation of debris and iris pigment granules. Prospective clinical trials comparing Nd:YAG versus argon LPI found that iridotomy closure occurred exclusively in eyes treated with argon laser (21–30% vs 0%), which had led to acute pupillary block in one eye with subsequent synechia formation that required trabeculectomy.^[88,89]^ However, iridotomy closure with Nd:YAG laser can occur especially in patients with uveitis^[90,91]^ and rubeosis.^[92]^ Repeat LPI can be performed at the same site or a new iridotomy can be created at another site.

#### Other complications

Rare complications include aqueous misdirection glaucoma, endothelial cell loss, recurrent herpetic keratouveitis, macular hole formation, retinal hemorrhages, and cystoid macular oedema.^[93,94,95,96,97,98]^

### Argon Laser Peripheral Iridoplasty (ALPI)

#### Indications

Argon laser peripheral iridoplasty (ALPI), also known as gonioplasty, utilizes low-energy argon laser to create circumferential burns on the peripheral iris. This induces contracture of the iris away from the anterior chamber angle, potentially resulting in the widening of the angle and the disruption of PAS. Early attempts in the late 1970s by Krasnov^[99]^ and Kimbrough^[100]^ to modify the peripheral iris had some success. Then the first ALPI procedure was proposed by Ritch in 1982 to treat medically unresponsive acute angle closure attack without distinguishing the underlying mechanisms.^[101]^

The primary indication for ALPI is detecting plateau iris in gonioscopy or imaging as the principal mechanism of acute angle closure.^[102]^ Numerous studies have reported cases where angle closure persists after LPI.^[103,104]^ This persistence may be attributed to the presence of an alternative underlying mechanisms. In cases of plateau iris, angle closure is primarily mediated by the crowded peripheral iris due to anterior rotation of the ciliary body, with or without a component of pupillary block. Consequently, LPI may prove ineffective or only partially effective, making the use of ALPI more sensible due to its mechanism of action, thinning and stretching of the peripheral iris. A study by Ritch et al demonstrated that ALPI effectively maintained open angles in 87% of eyes with plateau iris for the entire follow-up period (79 ± 8 months) following a single treatment. In the remaining eyes, the angle was reopened and maintained open following the second ALPI, eliminating the need for future surgeries.^[105]^ Conversely, other studies raised doubts about the long-term effectiveness of ALPI in plateau iris,^[106,107]^ since it only thins the superficial iris tissue without addressing the main pathology, the anterior rotation of the ciliary body.

Regardless the mechanism of acute angle closure, studies have shown that ALPI can be used as an initial treatment in some cases.^[108,109]^ While LPI serves as the classic initial intervention to reduce IOP in cases of acute angle closure due to pupillary block, the presence of corneal edema might hinder a clear view to safely perform the procedure.^[109]^ In such cases, ALPI was found to be a safe alternative in reducing IOP. Subsequently, as corneal edema subsides, definitive treatment with LPI can be pursued.^[108]^

ALPI may be used in nonophthalmic eyes, a developmental ocular disorder characterized by a normal eye but with a short axial length. In adulthood, nanophthalmic eyes are susceptible to angle closure because of high lens thickness to axial length ratio. ALPI has been demonstrated to effectively open the angle in some patients if appositional closure persists after LPI.^[100,110]^

#### Technique ${\;}^{\left[64,111\right]}$

Like LPI, pretreatment with topical pilocarpine is recommended to stretch the iris and increase access to the peripheral iris. Brimonidine or apraclonidine may also be administered to decrease the chance postoperative IOP spikes.

ALPI is performed using argon or diode laser applied to the anterior surface of the iris with low power (200–300 mW, incremental increase if no observed iris stromal shrinkage), long duration (0.3–0.5 seco), and large spot size (300–500 µm), aiming to induce contraction burns rather than perforation. There is a risk of pupillary dilation and distortion with closely spaced ALPI, therefore limiting the laser applications to no more than five to six evenly distributed shots per quadrant is recommended.

ALPI can be performed using either a direct or indirect technique. In the direct approach, laser energy is focused through the Abraham lens perpendicular to the peripheral iris, while the indirect approach employs a single mirror goniolens at a low angle of incidence toward the peripheral iris, while carefully avoiding the trabecular meshwork. The direct technique is generally easier to perform, while the indirect one offers better visualization of the angle. To avoid iris burns with direct application, the laser energy should be lower, and the spot size should be larger than the indirect approach.

#### Clear lens extraction

As the lens is a key, structural factor in development of relative pupillary block, lens extraction is an elemental surgical intervention in the management of primary angle closure disease. Ocular biometric studies using UBM have shown that eyes with PAC spectrum have several different characteristics as compared to normal eyes including significantly thicker lens.^[112,113,114]^ Removal of the crystalline lens deepens the anterior chamber angle, relieves iridotrabecular contact, and lowers IOP. Anterior segment changes following cataract surgery have been evaluated using UBM in several studies showing iris backward shift, increase in the ACD and the AOD.^[115,116]^ AS-OCT before and after phacoemulsification showed a significant increase in ACD and angle width in patients with preoperative open or narrow angles.^[117,118]^ Additionally, postoperative IOP reduction was proportional to the amount of angle opening, especially in narrow angle patients.^[117]^

Patients with evidence of cataract and those with a high degree of hyperopia could be candidates for lens extraction as a primary treatment of narrow angles. Presbyopia patients may also be good candidates, given improvements in presbyopia-correcting intraocular lens technologies. In the setting of an acute angle closure attack, cataract surgery could be challenging and technically difficult given the eye inflammation, corneal edema, shallow anterior chamber, and atonic poorly dilatable iris. The acute attack should be controlled with medications, with or without LPI first. After the eye is fully recovered, surgery can be performed.^[64,65]^

Lam et al^[119]^ compared the outcomes of LPI versus early lens extraction after abortion of acute angle closure attack in a randomized clinical trial. The results showed that the IOP was lower and remained more constant with lower number of medications through 18 months in the lens extraction group. Another randomized clinical trial by Husain et al^[120]^ compared the outcomes of LPI versus phacoemulsification in patients presenting with acute angle closure attack with coexisting cataract. The study showed superiority of lens extraction, and the two-year cumulative survival was 61.1% versus 89.5% for the LPI and cataract extraction, respectively (*P* = 0.034).

However, the choice between LPI and lens extraction in recently diagnosed PAC or PACG could be challenging in absence of a significant degree of cataract that may justify early phacoemulsification, a more invasive procedure, over LPI. The EAGLE study (effectiveness of early lens extraction for the treatment of PACG) was a multi-center, randomized clinical trial that aimed to answer this question.^[121]^ The study included newly diagnosed PAC or PACG eyes with IOP > 30 mmHg and clear lens (*N* = 419). The primary endpoints were patient-reported health status score assessed with the European Quality of Life-5 Dimensions questionnaire, IOP, and incremental cost-effectiveness ratio per quality-adjusted life-year gained 36 months after treatment. The study showed that the mean health status score was significantly higher (*P* = 0.005) and the mean IOP was significantly lower (*P* = 0.004) in the clear lens extraction group compared to the LPI group. Additionally, clear lens extraction was shown to be more cost-effective than LPI.

Despite the proven superiority of clear lens extraction over LPI in the EAGLE trial, a few points should be considered. First, the study only included patients with PAC with a minimum IOP of 30 mm Hg. Whether patients with PAC and IOP < 30 mmHg would benefit equally from clear lens extraction is therefore unclear. Additionally, clear lens extraction might be associated with severe intraoperative and postoperative complications. In the EAGLE trial, two participants had posterior capsule rupture, which is known to be associated with increased risk of poor visual outcomes. One participant developed aqueous misdirection glaucoma and another developed transient corneal oedema. Although the net effect of these surgical complications was small and the number of participants with irreversible vision loss was similar in both treatment groups, the risk of severe complications following clear lens extraction must be taken into account, especially if surgery is performed by less experienced surgeons.^[121]^

In chronic angle closure glaucoma, when IOP cannot be adequately controlled with medications in the setting of a patent LPI, lens extraction could be considered before proceeding to filtering surgery. A randomized clinical trial by Tham et al^[122]^ compared the effects of trabeculectomy versus lens extraction in patients with medically uncontrolled chronic angle closure glaucoma without cataract. The study showed that both interventions were effective in reducing IOP. Although trabeculectomy achieved less dependence on glaucoma medications, it was associated with more complications. With co-existence of extensive PAS, phacoemulsification can be combined with goniosynechialysis (using heavy viscoelastics, microforceps, or spatula) to break down the synechia and improve the IOP.^[123]^ However, cataract surgery alone without goniosynechialysis was shown to reduce the degree of PAS. This could be explained by either inaccurate preoperative gonioscopy due to the effect of a thick lens which does not allow posterior movement of the iris, or the possibility of the synechia release by repeated viscoelastic material instillation in the anterior chamber during cataract surgery to keep the anterior chamber formed.^[124]^

#### Filtration surgery

Glaucoma filtration surgery, including trabeculectomy and tube shunts, may be indicated in PACD. In chronic cases, with uncontrolled IOP on medications and progressive optic neuropathy, filtering surgery is employed.^[125]^ Given the presence of a thick lens and shallow anterior chamber, the filtering surgery may be considered after removing the lens. In acute angle closure attack, filtering surgery may be indicated when there is medical unresponsiveness, lack of laser availability, or signs of glaucomatous optic neuropathy already present.^[126]^ Trabeculectomy in angle closure is associated with high risk of failure and serious complications such as aqueous misdirection glaucoma, further shallowing of the anterior chamber and choroidal detachment.^[122,127]^ On the other hand, tube shunt implantation, although effective, could be challenging in the setting of a shallow anterior chamber.^[128]^

The outcomes of combined filtering surgery with cataract extraction (phacotrabeculectomy) versus phacoemulsification alone have been studied with mixed results. One study showed that phacoemulsification was superior in terms of deepening the anterior chamber.^[129]^ Another study showed that the two procedures were equal in terms of IOP control.^[130]^ A third study showed that combined procedure was superior for IOP control.^[131]^ A randomized controlled trial found that combined procedure was associated with more postoperative complications and progression of optic neuropathy compared to phacoemulsification alone.^[132]^ Given the lack of definitive evidence, the risks and benefits should be carefully evaluated for each case.

#### Minimally invasive glaucoma surgery 

Minimally invasive glaucoma surgery (MIGS) has become an important surgical approach for primary open angle glaucoma, yet its indications in angle closure glaucoma spectrum are less clear. IOP elevation in PAC and PACG is attributed to the mechanical obstruction of the angle by peripheral iris, rather than the dysfunction of the trabecular meshwork, which is often relieved by LPI or lens extraction. However, further studies have shown that the long-term persistence of iridotrabecular contact could damage the trabecular meshwork and compromise trabecular outflow in PACG by disruption of the blood–aqueous barrier, trabecular meshwork fibrosis, or damage of Schlemm's canal endothelium.^[133]^

Trabecular MIGS procedures target trabecular meshwork and Schlemm's canal pathway, and can relieve aqueous outflow resistance. Evidence suggests that cataract surgery in combination with goniosynechialysis and ab interno goniotomy is effective and safe in PACG. Dorairaj *et al* ^[134]^ examined PACG patients receiving cataract surgery and Kahook dual blade-assisted goniotomy and reported significant IOP and medication reduction after two years with 95% surgical success. Goniotomy using MVR blade as an adjunct with cataract surgery in PACG was also found to achieve similar results with lower cost compared with other micro-blades.^[135]^ Several retrospective and prospective studies on cataract surgery with gonioscopy-assisted transluminal trabeculotomy (GATT) in PACG reported significant IOP and medication reduction with high surgical success and low risk of complications even in advanced PACG.^[136,137,138]^

Trabecular stent implantation combined with cataract surgery was shown to be more effective than cataract surgery alone in PACG. Chansangpetch et al^[139]^ showed that cataract surgery and iStent implantation significantly improved surgical success and reduced the number of medications in patients with PACG after LPI as compared with cataract surgery alone. Chen et al^[140]^ reported that cataract surgery combined with iStent implantation had a higher success rate (87.5%) than cataract surgery alone (43.8%) in patients with PAC or PACG and cataract. Cataract surgery combined with Hydrus microstent implantation in PACG was shown to be effective in only one case report of a patient with mixed mechanism glaucoma.^[141]^

Subconjunctival stent MIGS or MIBS (minimally invasive bleb surgery) can also be used in combination with cataract surgery in PACG as a safer approach than trabeculectomy. Sng and colleagues^[142]^ reported that cataract surgery and XEN implantation significantly reduced medicated and non-medicated IOP as well as the number of medications in glaucomatous Chinese patients, 61% of the study population had PACG. Implant occlusion by iris was reported in only 1 eye among 31 eyes included in the study.

### Endoscopic Cyclophotocoagulation (ECP) and Endocycloplasty (ECPL) 

Endoscopic cyclophotocoagulation (ECP) is a cyclodestructive procedure developed by Uram in early 1990s.^[143]^ The goal was to minimize the adverse events of traditional cyclodestructive procedures while maximizing the efficacy of ablating the ciliary body of lower IOP. ECP utilizes a laser endoscope that comprises three fiber groupings: an image guide, a light source, and the semiconductor diode laser. Ciliary processes can be accessed through either a limbal or a pars plana approach. The limbal approach is typically recommended for patients undergoing ECP in conjunction with cataract surgery. This technology enables direct visualization of the ciliary epithelium, facilitating precise delivery of laser energy to the ciliary processes, with minimal damage to the underlying ciliary body and surrounding tissue.

Initial reports indicated the effectiveness of ECP combined with cataract surgery as a primary surgical intervention for open-angle glaucoma with a good safety profile.^[144]^ Later studies, encompassing various glaucoma types, including chronic angle closure with or without prior glaucoma surgeries, revealed a 34% reduction in IOP along with a decrease in glaucoma medications over one year. Treatment of at least 180º of ciliary processes was required to achieve reasonable IOP reduction.^[145]^

In a recent randomized clinical trial, the efficacy of combined phacoemulsification plus ECP was compared to phacoemulsification alone in PACG with coexisting cataract. The study included 48 eyes with a two-year follow-up. Results demonstrated that combined phacoemulsification with ECP led to a consistently lower IOP and reduced dependence on glaucoma medications compared to phacoemulsification alone. Statistically significant lower IOP measurements were observed at 1 month (*P* = 0.01), 12 months (*P* = 0.01), and 24 months (*P* = 0.04) postoperatively.^[146]^ Another retrospective study compared the outcomes of phacoemulsification combined with ECP alone versus ECP with goniosynechialysis in chronic angle closure. Both procedures effectively reduced IOP and glaucoma medications over one year. However, the reduction in IOP was more pronounced when ECP was combined with goniosynechialysis compared to ECP alone (42% vs. 26%).^[147]^

While the outcomes of ECP seem promising, the potential complications include uveitis, hyphema, cystoid macular edema, choroidal detachment, and a vision loss of two lines or more. More severe complications such as retinal detachment, choroidal hemorrhage, malignant glaucoma, hypotony, phthisis bulb, and progression to no light perception vision have been rarely reported.^[145,148]^

A novel modified technique of ECP, known as endocycloplasty (ECPL), is employed for managing angle closure secondary to plateau iris, combined with phacoemulsification. In ECPL, the laser energy is directed to the posterior aspect of the ciliary processes inducing shrinkage rather than destruction. The ablation results in the posterior retraction of the entire ciliary process resulting in angle widening and flattening the peripheral iris. Additionally, the procedure is expected to decrease aqueous production to some extent.^[149]^ Treatment of 270º of the ciliary body can be achieved through one incision. The endpoint of laser treatment is marked by adequate shrinkage and whitening of the ciliary process.^[149]^ Preliminary results from a retrospective study of 58 patients with plateau iris showed IOP reduction from 17.3 to 13.3 mm Hg, medication reduction from 1.7 to 0.7, and significant widening of the angle on gonioscopy and AS-OCT at three months (*P* < 0.01 for all). Adverse events were relatively mild and temporary.^[150]^

Another case series (*N* = 28) evaluated the outcomes of phaco-ECPL in patients with angle closure disease and coexisting cataract with longer follow-up (median 15 months). The results demonstrated significant IOP and medication reduction by the end of follow-up, and no serious sight-threatening complications were reported in any patient.^[151]^ The same author conducted a prospective randomized control trial to compare phaco-ECPL (mean follow-up 16 ± 8 months) versus phacotrabeculectomy (mean follow-up 19 ± 10 months) in PAC and PACG patients with visually significant cataract following LPI (*N* = 45). Both procedures were effective in lowering the IOP, however, the phacotrabeculectomy group exhibited a higher rate of complications (*P* = 0.011) and interventions (*P* = 0.047). These findings suggest that phaco-ECPL could be considered a safer and less invasive procedure for patients with cataracts and angle closure disease.^[152]^

##  SUMMARY

Gonioscopy is the most critical tool for diagnosis of narrow angle and classification of the angle closure disease spectrum, and it helps guide future management. New advancements in angle imaging, such as gonio-photographic systems, and the integration of machine learning into UBM and AS-OCT can provide valuable assistance alongside clinical gonioscopy with broader screening without an ophthalmologist present. These recent technologies can contribute to a more comprehensive understanding of angle-related pathologies, ultimately leading to more effective and personalized patient care.

Generally, in high-risk PACS, prophylactic LPI should be considered over observation. In PAC and PACG, lens extraction seems to offer better long-term protection than LPI. However, the management of primary-angle closure disease should be tailored based on the underlying mechanism. Those with pure pupillary block may derive the most benefit from LPI, whereas individuals with plateau iris should benefit from low-dose topical pilocarpine, ALPI, and ECPL. In patients with high LV, cataract surgery, with goniosynechialysis if necessary, can improve the angle anatomy, lower the IOP, and reduce the number of glaucoma medications. Filtering surgery may be required in cases of uncontrolled IOP with significant optic nerve damage, either following or combined with cataract surgery.

##  Financial Support and Sponsorship

None.

##  Conflicts of Interest

None.
